# The Dark Side of Motivational Practices in Exercise Professionals: Mediators of Controlling Strategies

**DOI:** 10.3390/ijerph17155377

**Published:** 2020-07-26

**Authors:** Frederico Zarazaga Raposo, David Sánchez-Oliva, Eliana Veiga Carraça, António Labisa Palmeira, Marlene Nunes Silva

**Affiliations:** 1 Universidade Europeia, 1500 Lisbon, Portugal; 2 Faculdade de Educação Física e Desporto, Universidade Lusófona de Humanidades e Tecnologias, 1749 Lisbon, Portugal; ecarraca@fmh.ulisboa.pt (E.V.C.); p126@ulusofona.pt (A.L.P.); mnsilva@fmh.ulisboa.pt (M.N.S.); 3 Faculty of Sport Sciences, University of Extremadura, 10004 Cáceres, Spain; davidsanchez@unex.es; 4 CIPER, Faculdade de Motricidade Humana, Universidade de Lisboa, 1495 Lisbon, Portugal; 5 Programa Nacional para a Promoção da Atividade Física, Direcção-Geral da Saúde, 1000 Lisbon, Portugal

**Keywords:** health & fitness settings, motivational strategies, self-determination theory, job pressures, controlling strategies, exercise professionals motivation, exercise promotion

## Abstract

According to the Self-Determination Theory, perceived job pressures can coerce professionals to develop more controlled motivations towards their work, and consequently increase the probability of using controlling motivational strategies. This study sought to analyze work-related motivations as mediators between two types of perceived job pressures: organizational constraints and perceptions of clients’ controlled motivation and the use of controlling motivational strategies by exercise professionals. Using a cross-sectional design, involving 366 exercise professionals (172 women), mediation paths were assessed following Preacher and Hayes statistical procedures. Models were adjusted for gender, work experience (years), and the internal tendency to feel events as pressuring. Organizational constraints were associated with lower autonomous motivation for work and the use of controlling strategies. Perceptions of clients’ controlled motivation were associated with work-related amotivation and the use of controlling strategies. Amotivation mediated the association between organizational constraints and controlling strategies. Overall, results support theoretical predictions and previous research, extending it to the exercise domain, highlighting the interplay between job pressures, work-related motivations, and the use of controlling strategies. The understanding of what influences exercise professionals’ motivation, and consequently the motivational strategies they use, is of paramount importance for exercise promotion and the benefit of those who seek their expert guidance.

## 1. Introduction

The preventative role of physical activity (PA) in health and quality of life [1], as well as in the treatment of more than 25 different chronic diseases [2] is well documented. Despite this, promoting long-term exercise adherence remains an important issue as adherence rates tend to be low, especially for those individuals who engage in some sort of PA but drop-out within the first six months [3]. Nevertheless, exercising at fitness facilities seems to be an emerging and growing trend [4]. Worldwide estimations point to around 187,000 health clubs, that serve approximately 151 million members. In the United States, total health-club members increased from 45.3 million in 2009 to 55.5 million in 2015 [4]. In Europe, namely in Portugal, 27% of those who engaged in exercise programs choose gym activities (vs. 15% in overall Europe) [5]. Furthermore, this seems to be a growing trend: the last report from the field points to an increase of 16% in Portuguese gym members between 2018 and 2019. However, attrition rates are equally elevated (58%) [6]. Simultaneously, estimates indicate that more than half of European adults are insufficiently active [3] and that physical inactivity increases noncommunicable diseases in 6–10% and premature death in approximately 9% of the inactive population [7]. Therefore, from a health and wellness perspective, analyzing and re-thinking motivational practices within the gym context may be of paramount importance, not only to continuously increase the number of people who choose this setting to exercise but especially, to ensure sustained engagement of those who already started [8].

### 1.1. Self-Determination Theory and the Role of Motivational Strategies

Prior research under the rationale of Self-Determination Theory (SDT) [9], has shown that PA can be autonomous regulated and, therefore, more easily maintained over time [10,11] supporting that higher levels of autonomous motivation foster higher degrees of intention toward exercising in the future [12]. Conversely, exercise professionals, coaches, parents, and health care practitioners can undermine PA, by acting in a pressuring or controlling way (i.e., undermining the basic psychological needs for autonomy, competence, and relatedness) [9,11,13]. Indeed, the type of motivational strategies (controlling or need supportive) used by health and fitness professionals can impact the subsequent experience of participants [10,14,15]. The use of these strategies has also been linked to maladaptive behaviors and affect in several domains [15,16]. An increasing body of research has highlighted the need to focus on professionals’ motivation and strategies they use with their clients, as they might facilitate, or not, behavior change [12,15,17], as well as create psychologically supportive contexts and identify behaviors associated with need support and need frustration to enhance emotional responses to exercise [18]. To properly address this, it becomes important to consider the antecedents of professionals’ motivation regarding their work, as that may influence the type of strategies used [8,15,19,20]. Little research has explored professionals’ own motivations and how it influences their practice, thus, gaining insight into professionals’ behaviors, beyond clients’ motivation and behavior, can be of extra value [8,15,21].

SDT proposes that there is a continuum in which motivational self-regulation varies. A basic distinction highlights the difference between autonomous and controlled motivations. Autonomous motivation involves the regulation of behavior with the experiences of volition, psychological freedom, and reflective self-endorsement, reflecting an internal perceived locus of causality. In contrast, controlled motivation involves experiences of pressures and coercion to think, feel, or behave in particular ways, reflecting an external perceived locus of causality. Both introjected (i.e., to be motivated to comply with a partially internalized contingency to gain pride and self-esteem, or to avoid feelings of guilt and shame) and external (i.e., to be motivated to engage in an activity, to obtain a reward or to avoid punishment) regulations, are controlled forms of motivation [9]. Autonomous and controlled motivation contrast with amotivation that represents a lack of intentionality and motivation, which can result from a felt lack of competence, of interest, relevance, value or a motivated defiance or resistance [22]. According to SDT, the social environment may or may not support the fulfillment of three basic psychological needs (i.e., autonomy, competence, and relatedness) leading to more autonomous or controlled types of motivation [23]. In this regard, the motivational strategies used by the professional are very important, as they may help to develop the client’s autonomous motivation to exercise or not. In this regard, two different interpersonal styles have been identified: need-supportive or controlling [24]. Need supportive professionals (e.g., teachers, health care practitioners, and exercise professionals) promote the volition of those they socialize, providing choice, offering a meaningful and realistic rationale when the choice is restricted, trying to understand the others perspectives. In contrast, controlling professionals seek to direct the thoughts, feelings, and behaviors of those with whom they interact. They may use overt pressuring strategies (e.g., controlling language and/or punishments), or other less evident techniques of manipulation, including conditional regard [25], guilt induction [26], and shaming [27]. Five separate controlling motivational strategies have been described in sport contexts [24]: (i) controlling use of rewards, that means providing tangible rewards as an incentive for completing a task, or for reaching certain performance standards; (ii) negative conditional regard, which is withholding love, attention and affection by those in a position of authority, when desired attributes or behaviors are not displayed by their subordinates; (iii) intimidation, that involves the display of power assertive strategies, designed to humiliate and belittle, such as verbal abuse and threats, yelling, threat, or use of physical punishment; (iv) excessive personal control, that involves the use of intrusive monitoring, and the imposition of strict limits, and (v) judging and devaluing, that means denying others’ thoughts and feelings, and treating them as objects that should be controlled in a way to obtain certain outcomes as winning or reaching a specific goal.

Despite all the evidence suggesting the association of need supportive strategies and contexts with higher wellbeing, better performance, behavioral persistence, and physical and mental health outcomes [10,14,19,22,28], indicating for the negative consequences of the use of controlling strategies for the professional I (for instance, contributing to their emotional exhaustion [29]. Several exercise professionals adopt a “no-pain, no-gain” instruction frame, often driven by the assumption that more controlling and pressurizing environments will be more effective in making class attendees work hard [12]. Furthermore, studies [15,30,31,32] in the educational domain, have shown that perceived job pressures (e.g., performance evaluation, time constraints, or pressure from the school administration), may lead teachers to develop controlled work-related motivations, increasing the odds of adopting controlling strategies. Conversely, there is also evidence that perceived organizational support and autonomous motivation for work are positively related to work satisfaction and negatively related to turnover intentions, which are instead predicted by controlled motivations [33].

### 1.2. Multiple Influences on Work Motivation and Potential Consequences 

Work motivation is under the influence of situational and dispositional factors [34]. Situational factors may include the way the work is divided, organized, and prescribed, as well as the quality of relationships with superiors, peers, subordinates, and clients. Dispositional factors can include personality traits such as optimism, deeply rooted causality orientations influencing reactions to work-related events, and circumstances (i.e., internal tendency to feel pressure and to perceive external events as pressuring). Accordingly, and to gain a deeper understanding of how different levels of work pressures/constraints may relate to the motivation of professionals and the type of strategies they use, a coherent framework distinguishing pressures from “above” (organizational), “below” (from the clients) and “within” (internal) has been developed [15,19,30]. In line with this, a recent review [35] divided antecedents of controlling strategies in three categories: contextual factors (socio-environmental factors and external pressure), perceptions of other’s behaviors and motivation, and personal factors. 

Contextual factors linked to pressures from “above” (e.g., from administrators or state standards) may be endorsed by professionals, especially if their professional interactions take place within a context where interpersonal power varies between interactants, which is common in education and several health settings when professionals routinely face job conditions steeped in accountability and responsibility for students/clients’ behaviors and outcomes. In such contexts, traditionally, a controlling motivational style might be culturally valued (e.g., teachers who use controlling instructional strategies are evaluated as more competent than teachers who use need-supportive ones) [19,30].

Pressures from “below” derive from professionals’ perception of students/clients’ decreased levels of self-determined motivation and can be understood as a request for more control and effort investment [19,30]. Research showed that teachers who perceive their students as more self-determined, tend to use strategies that maintain or facilitate it. In contrast, if they perceive low self-determination, they may use more controlling strategies [15].

The role of personal factors has also to be considered. Not all professionals are equally affected by pressures. Some professionals may have an internal predisposition to themselves and to interpret contextual influences as sources of pressure. Thus, pressures from “within” represent influences that arise from professionals’ own beliefs, values, and personality dispositions [19].

### 1.3. The Current Study

**Figure 1:**
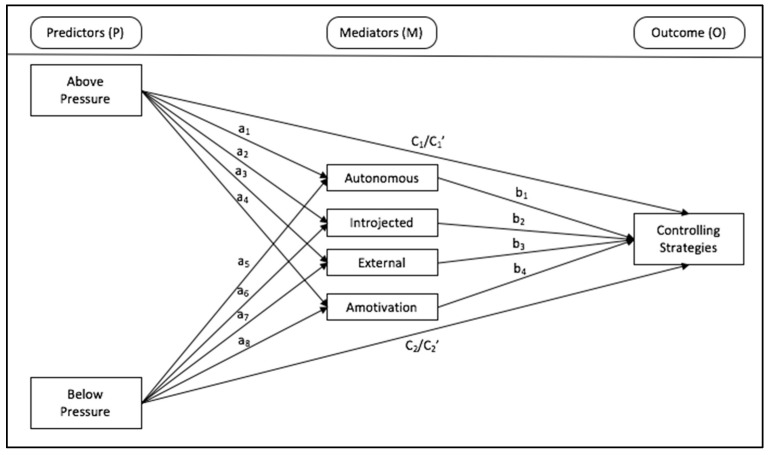
Hypothesized mediation model.

As demonstrated, the evidence points to the detrimental role of controlling motivational strategies, as potentially undermining long-term behavioral adherence and well-being, both from the client/student and professional standpoints [8,24,36]. Thus, the purpose of the present study is to explore potential predictors and mediators of the use of controlling strategies by exercise professionals working in health/fitness centers. The hypothesized model (Figure 1), is based on previous evidence pointing to the detrimental role that work pressures (at different levels) and less self-determined motivations to work, might play in the use of autonomy-supportive strategies, leading to the use of more controlling ones [15,35]. Thus, this study seeks to analyze the relationship between two different types of job pressures (i.e., organizational—“above”, and from the clients—“below”) and the reported use of controlling motivational strategies, while exploring the mediating role of different work-related motivations (i.e., reflecting autonomous and controlled-external, introjected-motivations, or even amotivation), while controlling for the role of internal pressures, gender, and years of work experience.

## 2. Materials and Methods

### 2.1. Participants

The current descriptive study was part of a larger cross-sectional survey [8], involving 366 exercise professionals (172 females, 193 males, 1 not specified), voluntarily recruited online, through two large contact lists from professional associations, currently working in health and fitness settings, all over the Portuguese country. Personal trainers, gym instructors, and exercise group class leaders, interacting directly with gym/health-club clients and responsible for the prescription and supervision of their exercise program, were included. Their ages ranged between 18 and 58 years (Mean = 34.16 years, SD = 6.37) and their work experience between 1 and 35 years (Median = 6; Mean = 7.7; SD = 5.8 years). Ethical approval was obtained from the research ethics council of the Faculty of Human Kinetics—University of Lisbon (Approval Number: CEFMH1/2014). Informed consent was obtained from all individual participants included in the study, prior to the online data collection.

### 2.2. Measures

Assessments included validated SDT-related psychosocial measures of the theoretical constructs under analysis. The psychometric properties of the Portuguese versions used in this study were acceptable and are briefly described in the results section, Table 1.

#### 2.2.1. Different Types of Perceived Job Pressures 

Different types of Perceived Job Pressures were assessed, according to Reeve’s distinction [19]:

1. Pressures from “above”

Pressures from “above” were analyzed using the Perceived Job Pressure Questionnaire [37]. The scale stem was adapted to the exercise domain as it was originally designed to assess physical education teachers’ perceptions. This scale includes 10 items and three dimensions reflecting three types of work-related pressures: perceived time constraints associated with PA classes (e.g., “I am sometimes rushing to complete my gym classes”); pressures associated with the organization (e.g., “My training methods are dictated by Gym policy”); pressures resulting from being evaluated based on clients/students’ performance (e.g., “I am held responsible for client performance standards”). A fourth subscale was added from the questionnaire Constraints at Work [30], measuring pressures associated with work colleagues (e.g., “You have to conform to your gym colleagues’ training methods”). Responses were reported on a 7-point scale, ranging from 1 (not at all true) to 7 (very true); some of the items were negative statements and therefore were reverse-scored before data analysis. Scores for each subscale were averaged and used as indicators of perceived job pressure from “above” in the hypothesized structural model.

2. Pressures from “below”

Pressures from “below”, (i.e., professionals’ perceptions of their client’s motivations for exercise), were measured with a previous adapted version of the Behavioral Regulation in Exercise Questionnaire (BREQ) [14,38]. The scale measures the perception of the professional in what concerns the different types of regulation for exercise displayed by the client: Amotivation (e.g., “My client cannot see why he/she should bother exercising.”); External (e.g., “My client exercises because his/her physician says he/she should”); Introjected (e.g., “My client exercises because he/she feels guilty when he/she doesn’t”); Identified (e.g., “My client exercises because he/she values the benefits of exercising”); Integrated (e.g., “My client exercises because he/she considers exercise a fundamental part of who he/she is”); and Intrinsic (e.g., “My client exercises because it is fun and pleasurable”). The scale includes 24 items covering the 6 subscales previously described. Responses were reported on a 5-point scale, ranging from 0 (not true for me) to 5 (very true for me). In line with the notion of an existing motivational continuum varying in the degree of self-determination, we used the “Relative Autonomy Index” (RAI) [39], calculated through the following formula: (2 * intrinsic) + identified—introjected—external—(2 * amotivation), as previously done [8,30]. Higher RAI scores reflected perceived greater self-determined (or autonomous) motivation from the client. 

3. Pressures from “within” 

Pressures from “within” (i.e., inherent tendency to understand the context as controlling and to feel pressure to act in a certain way), were assessed with the Index of Autonomous Functioning (IAF) [40]. The scale includes 11 items and three theoretically derived subscales assessing authorship/self-congruence (e.g., “My decisions represent my most important values and feelings”), interest-taking (e.g., “I often reflect on why I react the way I do”), and susceptibility to control (e.g., “I do things to avoid feeling bad about myself”). Responses were reported on a 5-point scale, ranging from 1 (“not at all true”) to 5 (“completely true”). Results from the first two subscales were reversed, to express pressures from “within”. Scores for each subscale were averaged and used in the hypothesized structural model. 

#### 2.2.2. Motivational Regulations for Work 

Motivational regulations for work were assessed using the Motivation at Work Scale [41]. This scale includes 12 items, divided into four subscales: Intrinsic (e.g., “Because I enjoy this work very much”); Identified (e.g., “I chose this job because it allows me to reach my life goals”); Introjected (e.g., “Because I have to be the best in my job, I have to be a “winner”), and External regulation (“Because this job affords me a certain standard of living”). An amotivation subscale derived from the Work Tasks Motivation Scale for Teachers (WTMST) [42] and consisting of three items (e.g., “I don’t know, I don’t see the relevance of this job”), was also included. Responses to all items were rated on a 7-point Likert scale ranging from 1 (not at all) to 7 (exactly). Similar to what was done in previous studies autonomous motivation was specified as a higher-order reflective latent variable with identified and intrinsic self-regulations as its lower-order latent indicators while controlled regulations (external and introjection) were still treated separately because they represent potentially dissimilar constructs with potentially differential consequences for behavioral adherence and well-being [43,44].

#### 2.2.3. Perceived Controlling Motivational Strategies

Perceived controlling motivational strategies used by exercise professionals were measured with an adapted version to the exercise domain of the Controlling Coach Behaviors Scale (CCBS) [24]. The stem was changed to reflect professionals’ perception of their practices instead of participants’ perceptions (i.e., “As an exercise instructor please indicate how much you agree or disagree with each statement”). It comprises five subscales: Controlling use of rewards (e.g., “I tend to use rewards/praise so that my clients train harder”); Negative conditional regard (e.g., “I am less supportive with my clients if they don’t train well”); Intimidation (e.g., “I shout at my clients to encourage them to complete the exercises”); Excessive personal control (e.g., “I try to interfere in aspects of my clients life outside the gym”); and Judging and devaluing (e.g., “I am very judgmental of my clients if they are not training well”). Responses were rated on a 7-point scale ranging from 1 (strongly disagree) to 7 (strongly agree). For this study, a total score was calculated, to classify the use of controlling strategies. 

### 2.3. Data Analysis 

All the analyses were performed using the software Mplus [45]. Different measurement models were tested to find the best model solution for each questionnaire. Confirmatory Factor Analysis (CFA) and Exploratory Structural Equation Modelling (ESEM) were performed. In the CFA analysis, items were restricted to load on their specific factor and the factors were permitted to correlate, whereas in the ESEM models the oblique target rotation was used [46]. All models were performed with the robust maximum likelihood estimator (MLR), available in Mplus software [45].

Secondly, factor scores derived from the retained measurement models were saved and used in the mediation analysis. Model fit was assessed based on the Chi-Square test, Comparative Fit Index (CFI), Tucker–Lewis Index (TLI), Root Mean Square Error of Approximation (RMSEA), and the Standardized Root Mean Square Residual (SRMR). Values greater than 0.90 for the CFI and TLI and smaller than 0.08 for the RMSEA and SRMR were considered acceptable [47]. 

The means, standard deviations, reliability (Cronbach’s alpha), and the correlation matrix were estimated. To test the main hypotheses, we conducted mediation analyses using the Mplus syntax created by Stride and colleagues [48], which are based on Hayes [49] process models. We used the bootstrapping resampling procedures (N = 10,000) to compute 95% bias-corrected confidence intervals (95% BcCI). Indirect effects were considered significant if the 95% BcCI did not include zero. This model was estimated using the maximum likelihood (ML) estimator as MLR with bootstrapping is not yet available in Mplus. For mediation purposes, Mplus is particularly well-suited, as it can handle models with multiple independent/dependent variables, mediators in series or in parallel, latent variables, and non-normal dependent variables [48].

As presented in Figure 1, the model tested included both pressures from “above” and “below” as independent variables; autonomous, introjected, external regulations, and amotivation as mediators; and controlling strategies as the dependent variable. Specifically, we analyzed the direct effect of both types of pressure on the different types of motivations and controlling strategies, as well as whether the associations between job pressures and controlling strategies were mediated by the different types of motivations. The analyses were adjusted for gender, work experience (years), and the internal tendency to feel events as pressuring.

## 3. Results

### 3.1. Preliminary Analysis 

The fit statistics for the measurement models for each measure are presented in the Table A1. In all cases, the ESEM models showed a better degree of fit to the data (greater values in CFI and TLI and lower values in RMSEA, SRMR) when compared to the CFA, as well as acceptable factor loadings (significative target-loadings and greater 0.40 and cross-loadings lower than target-loadings). As displayed in the Table A1, the ESEM representation of all measures showed an acceptable degree of fit to the data (CFI and TLI > 0.90, RMSEA < 0.06, and SRMR < 0.08), and the ESEM model of each scale (factor scores) were used as the retained solution in the following analysis.

The means, standard deviations, bivariate correlations, and reliability coefficients for all variables used in the present study are reported in Table 1. Bivariate correlations showed that perceived pressures from “above” were negatively associated with autonomous motivation and positively associated with controlling strategies (*p* < 0.001), whereas pressures from “below” were positively associated with amotivation and controlling Strategies (*p* < 0.001). Introjected and external regulations and amotivation were positively associated with the use of controlling strategies (*p* < 0.005), while autonomous motivation was not.

### 3.2. Main Analysis

Table 2 presents both the direct and indirect effects between study variables. The model was designed to analyze the effect of both job pressures (“above” and “below”) on controlling strategies, via different types of regulation towards work (autonomous, introjected, external, and Amotivation). Pressures from “above” were directly and positively associated with controlling strategies (β = 0.203; *p* < 0.001), but were not mediated by any type of motivation for work: Autonomous regulation (β = 0.009; 95% BcCI [−0.008, 0.025]), introjected regulation (β = −0.003; 95% BcCI [−0.021, 0.015]), external regulation (β = 0.000; 95% BcCI [−0.006, 0.006]), or amotivation (β = −0.007; 95% BcCI [−0.030, 0.016]). Pressures from “below” were directly and positively associated with controlling strategies (β = 0.180; *p* < 0.001), and indirectly via amotivation (β = 0.040, 95% BcCI [0.003, 0.077]). However, the other types of motivation were not significant mediators: Autonomous Regulation (β = 0.002, 95% BcCI [−0.006, 0.011]), introjected regulation (β = 0.015, 95% BcCI [−0.006, 0.037]), or external regulation (β = 0.000, 95% BcCI [−0.006, 0.006]).

## 4. Discussion

The main goal of this study was to explore the role of different types of work-related motivational regulations as mediators of the association between different types of job pressures and the use of controlling motivational strategies in a sample of exercise professionals. Overall, the results confirm the relevance of perceived pressures from “above” (i.e., organizational) and “below” (i.e., perceptions of client’s self-determination level) as correlates of the use of controlling strategies, further suggesting that professionals’ amotivation towards work might partly explain the positive associations between perceived “below” pressures” and the use of controlling strategies. In contrast, and against expected, neither autonomous nor controlled motivations appear to mediate these associations.

Consistent with prior evidence, mainly from physical education and sports fields [15,30,35,50], both types of job pressures (“above” and “below”) were positively related to the reported use of controlling strategies. Thus, decreasing both could be important to reduce the use of controlling strategies, and subsequently increase basic psychological needs’ satisfaction [15], creating an adaptive interpersonal environment to participants [51] and, ultimately, exercise adherence [10] and persistence [52]. 

Organizational constraints (i.e., pressures from “above”) over exercise professionals may be materialized in obligations to follow a gym methodology, colleagues’ expectations and demands, pressures to maximize clients’ performance and results, administrative pressures (e.g., gym managers, fitness directors), performance evaluations (e.g., number of sales, number of participants in the classes, number of personal training lessons delivered), and administration of rewards [35]. Our findings reinforce the idea that organizational policies have an important role in the motivational style selected and used by exercise professionals [19,30,35,50]. This perspective is complemented by Reeve et al. [53], who proposed that controlling policies may lead professionals to believe that a controlling style is the norm in their contexts, and this may encourage them to use controlling strategies to motivate their clients. The present study also showed that pressures from “above” were negatively related to professionals’ autonomous regulation for work. Thus, diminishing organizational pressures might facilitate autonomous regulation towards work, as theoretically predicted [35,54], even in more experienced professionals [55].

Exercise professionals’ amotivation and controlled regulations (external and introjected) towards work were related to the higher reported use of controlling strategies. This is particularly relevant, given that these findings are in line with previous literature in the education [15,19,30,50], coaching [55,56], and exercise domains [8,57], indicating that lower self-determination towards work is positively associated with the use of controlling strategies and emotional exhaustion. Work-related motivation has also been linked with employees’ performance and wellbeing: if motivated by controlling reasons, professionals tend to express more work-related exhaustion, burnout, and turnover, as well as less work satisfaction, commitment, and performance [54]. It might be that when subject to a more controlled motivation (implying more pressure on themselves), exercise professionals also end-up putting more pressure on their clients (by using more controlling strategies); an avenue explored in other domains that also seems to apply to this specific population and setting [15,19,58]. Conversely, we found no significant relationship between autonomous motivation for work and the use of these strategies. This lack of association instead of the expected negative association as the theory would predict may imply that having an autonomous motivation for work is not protective against the use of controlling strategies. This finding highlights the relevance of studying and intervening on the “dark side” of motivation and its antecedents (e.g., perceived pressures). Indeed, besides creating autonomy-supportive work contexts, organizations might also need to focus on diminishing pressures and avoiding the development of controlling motivations towards work. As previous studies have shown, autonomous and controlled motivations are not simply opposites, they are orthogonal constructs, and can both be present at the same time [59]. 

Despite the direct associations found between work-related motivations and the other constructs under analysis (job pressures and strategies used), controlled regulations did not mediate the association between pressures from “above” and the use of controlling strategies. This finding contradicts prior research indicating that the effects of external pressures on controlling behaviors were mediated by teachers/coaches’ self-determined motivation [15,30,31,32]. The existence of direct relationship but not mediation may imply that the deleterious effect of pressures from “above” might be rather directly reflected on the use of controlling strategies or through other variables (e.g., need frustration). 

Concerning the association between pressures from “below” and the use of controlling strategies, a different scenario was found. In this case, being amotivated for work was a significant mediator (explaining 61% of the total effect). Autonomous, introjected, and external motivations were not significant. Pelletier, Séguin-Lévesque, and Legault [30] investigated the mediating role of self-determination towards work in a school context, showing that the relation between perceived students’ motivation and teachers’ behavior was mediated by teachers’ motivation. The authors concluded that students who lack motivation may be perceived as aversive, and they may make teachers feel incompetent or disliked by the student, leading them to assume a more controlling behavior and to have less desire to spend time with such students. This type of pressure may create an effect of “motivation contagion”. That is, by perceiving their clients as less motivated to exercise, professionals’ own motivation may start to be affected as their frustration increases (e.g., fostered by feelings of incompetence, rejection, and disappointment). In the opposite way, when professionals feel that clients are engaged in the sessions a positive cycle occurs: they are more likely to use strategies that maintain or facilitate clients’ self-determination, consequently, they may increase their own pleasure and fulfillment for a fulfilling job [15,28]. This mediating effect of exercise professionals’ self-determination between their perception of clients’ self-determination and the motivational strategies they use has already been described before [15,28,56,58,60,61]. 

Work-related amotivation seems to be an important mediator of professionals’ use of controlling strategies; therefore, it seems crucial to prevent its development. Prior reviews have shown that promoting autonomous regulation is of little additional value, once the professional is amotivated to work [35]. Furthermore, it has been previously argued that need-supportive behaviors require more investment from the exercise professionals than controlling behaviors, which can explain why under pressure and lack of motivation they may opt for these kinds of strategies [62]. If they are not self-invested (autonomously motivated) in their work, chances are that they do not invest positively in their clients as well.

## 5. Limitations

This study has limitations that should be addressed in future research. First, the study is cross-sectional in nature, and although our modeled relations are theoretically based, we cannot exclude the possibility of reverse causality between the outcome variables (i.e., strategies used by exercise professionals) and the putative mediators (i.e., motivation to work). Another limitation concerns the use of self-reported instruments to assess the motivational strategies used by professionals. Alternative measures could be considered such as the use of independent observer ratings to assess the frequency of use of each strategy, avoiding potential biases due to social desirability. Furthermore, even though all questionnaires used in this study are internationally validated instruments, some of them are yet in the process of being formally validated to the Portuguese population. Still, psychometric properties were assessed to partly overcome this limitation.

## 6. Conclusions

The present study supports the detrimental role of perceived job pressures via its prejudicial association with the type of motivational strategies used by exercise professionals. When gym managers pressure professionals by emphasizing short term outcomes (e.g., number of “selling’s”/personal training sessions per week/month; weight-loss), this may not be tolerable in the long term, undermining professionals’ wellbeing and potentially creating feelings of job insecurity, which in turn is one of the predictors of controlling behaviors [63]. In line with recent literature and evidence, improving the work context in a gym or health-club involves allowing exercise professionals to gain competencies and/or feel confident, feel free to experiment, and initiate their own behaviors without feeling pressured or coerced, and feel respected both by supervisors and peers. These policies or practices are likely to fulfill basic psychological needs and promote autonomous motivation, wellbeing, and high-quality performance. On the contrary, the thwarting of these needs tends to promote controlled motivation or amotivation, burnout, and poorer quality of performance [35,50,51].

Exercise professionals’ perception of their clients’ self-determined motivation may be an important trigger for their adoption of a need-supportive or controlling interpersonal motivational style. It is, therefore, important to provide the professionals with the understanding that need-supportive coaching is in fact the more adaptive way to foster motivation, even amongst exercisers/clients with low self-determined motivation [35], and educating them about the potential consequences of using controlling strategies (i.e., promotion of clients’ controlled motivation and amotivation; negative consequences for themselves, and increasing the risk of burnout) [18]. Exercise professionals need to be trained on how to become less controlling and more autonomy-supportive, to create supportive environments [57]. This is an effort that needs to be acknowledged and facilitated by health and fitness organizations, not only to continuously increase the number of people who choose this setting to exercise but especially, to ensure sustained engagement of those who already started.

## Figures and Tables

**Table 1:** Descriptive and correlational analysis among the study variables.

	M	SD	α	1	2	3	4	5	6	7
1. Pressures from “Above”	2.79	0.93	0.79	-						
2. Pressures from “Within”	2.26	0.51	0.71	0.02	-					
3. Pressures from “Below”	1.89	0.90	-	0.14 **	0.04	-				
4. Autonomous Regulation	5.75	0.90	0.84	−0.16 **	−0.18 **	−0.07	-			
5. Introjected Regulation	4.65	1.29	0.66	−0.00	−0.06	0.10	0.51 **	-		
6. External Regulation	3.56	1.33	0.84	−0.01	−0.07	0.00	0.36 **	0.50 **	-	
7. Amotivation	1.28	0.74	0.86	0.00	0.120 *	0.16 **	−0.25 **	−0.01	−0.01	-
8. Controlling Strategies	3.07	0.92	0.83	0.26 **	0.12 *	0.29 **	−0.08	0.20 **	0.11 *	0.27 **

Note: * *p* < 0.05. ** *p* < 0.01. α = Cronbach’s alpha coefficient. The reliability score of perceived self-determined motivation were: Intrinsic Motivation: 0.79; Identified regulation = 0.65; Introjected regulation = 0.66; External regulation = 0.84; Amotivation = 0.86.

**Table 2:** Mediation results in controlling strategies as dependent variables.

Predictors (P)	Mediators (M)	Total Effect (C)	P → M (A)	M → O (B)	Direct Effect (C’)	Indirect Effect
Pressures from “Above”		0.201 **			0.203 **	−0.001 (−0.037, 0.034)
Autonomous			−0.144 **	−0.060		0.009 (−0.008, 0.025)
Introjected			−0.014	0.197 **		−0.003 (−0.021, 0.015)
External			−0.010	0.023		0.000 (−0.006, 0.006)
Amotivation			−0.032	0.225 **		−0.007 (−0.030, 0.016)
Pressures from “Below”		0.238 **			0.180 **	0.058 * (0.012, 0.103)
Autonomous			−0.042	−0.060		0.002 (−0.006, 0.011)
Introjected			0.077	0.197 **		0.015 (−0.006, 0.037)
External			−0.005	0.023		0.000 (−0.006, 0.006)
Amotivation			0.178 **	0.225 **		0.040 (0.003, 0.077)

Note: * *p* < 0.05. ** *p* < 0.01.

## Appendix A

**Table A1:** Measurement models.

	MLR x*^2^*	*p*	*df*	CFI	TLI	SRMR	RMSEA [90% CI]
Pressures from “Above”							
4-Factors CFA	256.655	0.000	48	0.823	0.756	0.074	0.109 (0.096, 0.122)
4-Factors ESEM	47.396	0.000	24	0.973	0.923	0.027	0.052 (0.029, 0.073)
Pressures from “Below”							
6-Factors CFA	884.829	0.000	237	0.851	0.826	0.079	0.086 (0.080, 0.093)
6-Factors ESEM	393.297	0.000	147	0.930	0.868	0.026	0.068 (0.060, 0.076)
Pressures from “Within”							
3-Factors CFA	179.754	0.000	41	0.803	0.735	0.081	0.096 (0.082, 0.111)
3-Factors ESEM	43.552	0.012	25	0.974	0.942	0.026	0.045 [0.021, 0.067]
Motivational regulations for work							
5-Factors CFA	349.364	0.000	80	0.884	0.847	0.073	0.096 (0.086, 0.106)
5-Factors ESEM	99.969	0.000	40	0.958	0.905	0.023	0.064 (0.048, 0.080)
Controlling Behaviors							
5-Factors CFA	164.206	0.000	67	0.898	0.862	0.053	0.063 (0.051, 0.075)
5-Factors ESEM	39.870	0.132	31	0.991	0.973	0.018	0.028 (0.000, 0.051)

Note: x*^2^* = Chi-Square test; *df* = Degree of freedoms; CFI = Comparative Fit Index; TLI = Tucker-Lewis Index; RMSEA = Root Mean Square Error of Approximation; SRMR = the Standardized Root Mean Square Residual. CFA = Confirmatory Factor Analysis; ESEM = Exploratory Structural Equation Modelling.
